# Relationship between Adherence to the Mediterranean Diet and Body Composition with Physical Fitness Parameters in a Young Active Population

**DOI:** 10.3390/ijerph17093337

**Published:** 2020-05-11

**Authors:** Samuel Manzano-Carrasco, Jose Luis Felipe, Javier Sanchez-Sanchez, Antonio Hernandez-Martin, Ivan Clavel, Leonor Gallardo, Jorge Garcia-Unanue

**Affiliations:** 1 Investigación en Gestión de Organizaciones Instalaciones Deportivas Research Group, Faculty of Sport Sciences, University of Castilla-La Mancha, 45071 Toledo, Spain; samuel.manzano@uclm.es (S.M.-C.); antonio.hmartinsan@uclm.es (A.H.-M.); leonor.gallardo@uclm.es (L.G.); jorge.garciaunanue@uclm.es (J.G.-U.); 2 School of Sport Sciences, Universidad Europea de Madrid, 28670 Madrid, Spain; javier.sanchez2@universidadeuropea.es; 3 Department of Physical Education and Sport, Faculty of Sports Sciences and Physical Education, University of A Coruña, 15008 A Coruña, Spain; clavel.ivan@gmail.com

**Keywords:** nutrition, physical activity, lifestyle, obesity, adolescents, children

## Abstract

The current study aimed at analyzing the relationship between body composition, adherence to the Mediterranean diet (MD), and physical fitness (PF) in a young active population. A total of 1198 athletes (boys = 875; girls = 323) enrolled in different municipal sports schools participated in this study. Data on adherence to the MD (KIDMED questionnaire), anthropometric measurements, and PF (20 m shuttle run test, handgrip strength, vertical jump and forced spirometry) were collected. Results show that the pubertal boys had a higher score in the KIDMED test than the prepubertal ones (+0.38, *p* = 0.28). Moreover, boys with better adherence to the MD had significantly higher results in handgrip strength (+12.20 regarding low MD group and +9.13 regarding medium MD group, *p* < 0.05), as well as in forced vital capacity (FVC) (+0.66 regarding low MD group and 0.29 regarding medium MD group, *p* < 0.05). No differences were found in the girls. Finally, the result of the KIDMED test is a variable with a positive and significant relationship with cardiorespiratory fitness, along with the FVC, percentage of fat mass, and performance in the vertical jump (*p* < 0.05). It is concluded that adherence to the MD could show a relationship with various PF variables in boys and could be a predictor of cardiorespiratory fitness in both cases.

## 1. Introduction

Childhood is one of the most important periods of life because this is the time when life habits are established, and a large number of physiological and psychological changes happen [1]. Overweight and obesity in childhood and adolescence are associated with adverse health consequences throughout the later stages of life [2]. Moreover, the prevalence of overweight and obesity in children and adolescents has increased in recent years, leading to increased cardiovascular risks and metabolic diseases [3].

Healthy lifestyle interventions are the most common strategies for children and adolescents with obesity. Furthermore, habits acquired at these early ages can be a good predictor of health, preventing chronic non-communicable diseases. Some of the habits that lead to an active and healthy lifestyle are practicing daily Physical Activity (PA) as well as healthy nutrition patterns [4]. Currently, scientific evidence has shown the efficacy of PA and physical exercise in improving indicators of physical, psychosocial, and cognitive health in schoolchildren [5]. Exercise in the appropriate dose causes a specific adaptive response [6] that manifests itself, among other variables, in an improvement in the components of physical fitness (PF), establishing a direct relationship between PF and the state of health at early ages [7]. Likewise, the promotion of PF in childhood has a protective effect on health during adulthood [8]. Scientific evidence has shown that each component of PF has a positive effect on the health status of young people [9]. Thus, higher levels of cardiorespiratory fitness along with muscular strength and body composition during childhood and adolescence is associated with a healthier cardiovascular profile and a lower risk of death later in life [10]. Improvements in muscular fitness and speed/agility have a positive impact on bone health, cardiovascular levels and the muscular fitness itself [11]. Therefore, following and controlling balanced eating habits together with a good level of PF are important factors for present and future health [10,12].

The Mediterranean diet (MD) [13] has been considered to be a model of healthy diet, demonstrating a reduction in cardiovascular mortality in the population adhering to this eating pattern [14]. In its original definition, the traditional MD is characterized by a high intake of vegetables, legumes, fruits, nuts, and cereals, a high intake of olive oil but a low intake of saturated lipids, a moderately high intake of fish, a low-to-moderate intake of dairy products, a low intake of meat and poultry, and a regular but moderate intake of ethanol, primarily in the form of wine and generally during meals [15]. This MD has been associated with low prevalence and/or incidence with various diseases, including type II diabetes, hypertension, cardiovascular disease, and certain cancers, all of them associated with being overweight [16,17]. Finally, it seems that there is an association between these two healthy habits, since previous studies have revealed that in children, poor diet patterns correspond with excessively sedentary lifestyles, while healthy eating habits are associated with the greater practice of PA [18].

Hence, it is evident in the literature that PF and adherence to the MD, especially during childhood, are determining factors in the prevention of chronic non-communicable diseases, and there seems to be a certain association between them. These represent good indicators of healthy lifestyles, and it is essential to acquire them at an early age since healthy lifestyles are a protecting factor of health in adulthood. Therefore, the purpose of this study was to analyze the relationship between eating habits, body composition, and PF in a young active population.

## 2. Materials and Methods

### 2.1. Sample

The present study is descriptive and cross-sectional. The participants were 1198 athletes (875 boys and 323 girls) from six to 17 years old, enrolled in different municipal sports schools in Castilla-La Mancha (in central Spain). The sample was selected using nonprobability sampling. However, although we used this type of sampling, all athletes from the selected municipal sports schools were invited to participate. The participants included in the study were those who, after being previously informed about the objective of the study and the different tests, were authorized through an informative document signed by their respective parents or legal guardians. As an exclusion criterion, we did not include those who were exempt from participation in a training session. The athletes were evaluated individually on a single occasion. Furthermore, the Marshall and Tanner test was used to control the pubertal status of the participants [19,20]. A total of 527 boys and 230 girls were classified as prepubertal (stage I), and 348 boys and 93 girls were classified as pubertal (stages II and III). In addition, hours per week of planned out-of-school PA were registered.

This research was carried out in compliance with the standards of the Declaration of Helsinki (2013 revision, Brazil) and following the guidelines of the European Community for Good Clinical Practice (111/3976/88, July 1990). This study was approved by the Bioethics Committee for Clinical Research of the Virgen de la Salud Hospital in Toledo, Spain (REF: 508/17042020).

### 2.2. Procedures

#### 2.2.1. Eating Habits

The existence of possible eating disorders and adherence to the MD was determined using the KIDMED questionnaire. This test, previously validated, contains a 16-question test where the index varies from 0 to 12 [21]. Questions that present negative aspects in relation to the MD are scored with a value of −1, and those with positive aspects with +1. The KIDMED index is the sum of all values from the administered test is categorized into three different levels—(1) low adherence (very low-quality diet, 0–3); (2) medium adherence (improvement of the diet is needed, 4–7); (3) and high adherence (optimal adherence to the MD, 8–12) [21].

#### 2.2.2. Anthropometric Measurements

Each individual athlete had an anthropometric evaluation. For this, a portable segmental analyzer of multifrequency body composition (Tanita MC-780, Tanita Corp., Tokyo, Japan) was used to measure weight (kg), fat mass (%), and lean mass (%). The Body Mass Index (BMI) was calculated with the weight (in kilograms) divided by the squared height (in meters). The height (m) was measured with a height rod (Seca 214, Hamburg, Germany). All participants were evaluated with clothes and without sports.

#### 2.2.3. Physical Fitness

The different parameters of PF were assessed following the protocols of the ALPHA health-related fitness battery [22]. Cardiorespiratory fitness was evaluated with the 20 m shuttle run test (20 mSRT). This maximum and progressive test measures the maximum cardiorespiratory fitness [23]. Participants were required to run, in a straight line, between two lines distanced 20 m apart and maintaining speeds provided by acoustic signals from an audio speaker with Bluetooth technology. According to the protocol by Léger et al. [24], the initial speed was 8.5 km × h^−1^, which was increased by 0.5 km × h^−1^ each min (1 min = one stage). The test was finished when the athletes failed to reach the end lines before the audio signal on two occasions or when stopped because of fatigue. Athletes were allowed to perform the test once. 

Muscular fitness was evaluated using the handgrip strength and vertical jump. Upper-body muscular strength (kg) was valued through the handgrip strength test using a hand dynamometer with adjustable grip (TKK 5001 Grip A., Tokyo, Japan). Participants were given a short demonstration and verbal order for the test, and the dynamometer was regulated according to the child’s hand size. Subjects had to continuously tighten for 2 s with the elbow position in full extension. The test was repeated twice (right hand and left hand alternately). The best score of the two attempts for each player was chosen to the nearest 1 g [22]. 

Lower-body muscular strength was calculated by means of the vertical jumping test. Maximal vertical jump height was assessed to the nearest 0.1 cm during a countermovement jump (CMJ) with arm swing using photoelectric cells consisting of two parallel bars (Optojump, Microgate, Bolzano, Italy), which measure flight time taken as the duration between take-off and landing. Each participant was familiarized with the CMJ test prior to data collection. Participants were instructed to jump as high as possible, with a rapid, preparatory downward eccentric action while arms were freely able to be moved. All participants completed three jumps separated by 1 min of passive recovery with the highest jump taken as the final outcome measure. The CMJ arm swing test is a valid and reliable field test for the assessment of muscular fitness [25]. These results of PF variables were transformed into standardized values of percentiles according to age and sex [26,27].

Finally, respiratory capacity was recorded through forced spirometry. This test consists of a physiological test that measures how a subject inhales or exhales volumes of air as a function of time [28]. Each of the athletes performed a maximum inspiration using a spirometer, no more than two seconds of apnea, and a maximum expiration until there is no air left in the lungs. The most important aspects of spirometry are the forced vital capacity (FVC), which is the volume delivered during an expiration made as forcefully and completely as possible starting from full inspiration, and the forced expiratory volume (FEV) in one second, which is the volume delivered in the first second of an FVC maneuver [28]. Moreover, other spirometric variables were also assessed. Two measurements were implemented for each athlete, and the best measurements was preserved.

### 2.3. Statistical Analysis

Results are presented as means (M) ± standard deviations (SD). The Kolmogorov–Smirnov test showed a non-normal behavior of the variables; therefore, non-parametric tests were used. Firstly, the U Mann–Whitney test was performed to compare KIDMED scores based on the pubertal status. After that, the Kruskal–Wallis H test was performed to compare to compare the different body composition and PF variables between the three MD adherence groups. Where differences were identified, post-hoc pairwise comparisons were performed using Dunn–Bonferroni tests available on SPSS v21.0. (SPSS Inc, Chicago, IL, USA). Furthermore, Cohen’s d was calculated to assess the effect size of differences [29]. Finally, the relationship between KIDMED score and the main PF parameters: 20 mSRT (min), handgrip (kg), CMJ (cm), and FVC (l) was evaluated by linear regression analysis. Four models were estimated, one for each main PF parameters, including the rest of the PF variables, sex, age, BMI, weekly PA (mins/day), fat mass (%), and KIDMED score as independent variables. Because sex, age, and BMI are included as control variables, 20 mSRT, handgrip, and CMJ were included as min, kg, and cm, respectively, and not as percentile (i.e., the regression lines fit better this way). The objective the estimations is not to get the tightest model with fewer variables but to analyze the existing relationship between all of them, including control variables in the model. Therefore, linear regression by the ordinary least square method was estimated. The model did not present problems of heteroscedasticity or normality of errors of residuals. Moreover, variance inflation factor (VIF) was calculated to adjust the regression and prevent multicollinearity problems. The statistical significance was established as *p* < 0.05.

## 3. Results

**Figure 1:**
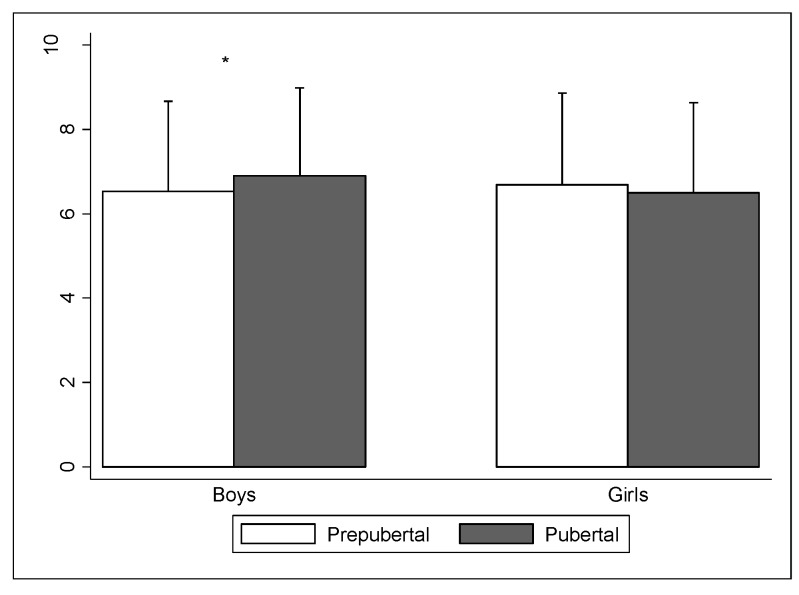
Differences between pubertal status. * Differences between prepubertal and pubertal groups, *p* < 0.05

The percentages of adherence to the MD not shown significant differences between boys and girls. A total of 7.1% of the boys (*n* = 62) had a very low diet quality, 57.6% (*n* = 504) needed to improve their diet, and 35.3% (*n* = 309) had optimal adherence to the MD. On the other hand, 7.1% of the girls (*n* = 23) had a very low diet quality, 59.8% (*n* = 193) needed to improve the diet, and 33.1% (*n* = 107) had good adherence to the MD. The comparative analysis between prepuberal and pubertal (Figure 1) in total KIDMED score only reveal significant differences in the case of the boys, but with trivial effect size (−0.37, *p* = 0.28, ES: 0.17).

Table 1 shows the differences between adherence to the MD groups in each sex. The high MD group presented significantly higher values in handgrip strength compared to low (+12.20, *p* = 0.007, ES: 0.42) and medium adherence to the MD (+9.13, *p* < 0.001, ES: 0.28). The cardiorespiratory variables show significant growth in each MD group. Medium MD showed better values than low MD in all cardiorespiratory values except FEV_1_/FVC (*p* < 0.05, ES: 0.34 to 0.46), as is the case with high MD compared to low MD (*p* < 0.05; ES: 0.57 to 0.62). The high MD group also presented higher values than medium MD group in FVC (+0.29, *p* = 0.001, ES: 0.28), FEV_1_ (+0.22, *p* = 0.002, ES: 0.25) and Forced Expiratory Flow (FEF_25–75_) (+0.19, *p* = 0.042, ES: 0.18). However, there are no significant differences between groups in the case of girls.

Finally, Table 2 shows the relationship between main PF fitness parameters, KIDMED score, and other control variables. All models showed an important fit. KIDMED score only has a significant influence in 20 mSRT (*p* = 0.032). Age showed a positive and significant influence in all PF parameters (*p* < 0.01), and fat mass showed a negative and significant influence (*p* < 0.05). BMI showed a significant and negative relationship with 20 mSRT (*p* < 0.05) and a positive relationship with handgrip and FVC (*p* < 0.01). Finally, all PF variables have a significant and positive relationship (*p* < 0.05), except 20 mSRT and handgrip.

## 4. Discussion

This study examined the relationship between adherence to the MD, body composition and PF in a cohort study of 1198 young active population. The data obtained from the KIDMED questionnaire in children (low 7.1%, moderate 57.6% and high adherence to the MD, 35.3%) are superior to the studies carried out in Mediterranean countries [30]. Results showed a positive tendency to sustain and improve patterns associated with this type of diet, given that 92.2% of our population shows moderate or optimal adherence to the MD. Therefore, a more active lifestyle is related to a higher score in the KIDMED [31,32]. Besides adherence to good eating habits, no differences were found according to gender, as previously evidenced [30], caused by context and similar lifestyle in both genders [33]. However, there were significant differences between prepubertal and pubertal children. Notably, prepubertal children show a distinct metabolic profile than pubertal or adolescent subjects and respond differently to metabolic challenges [34]. Therefore, these findings may be due to the combination of different factors in women, such as the ideal of thinness or the exaltation of the body, the stigmatization of obesity, the evolution of gender stereotypes, and the aggressiveness of marketing [35]. In addition, it is important highlight the importance of parental support and the influence of the school environment for the generation of healthy habits [36]. 

When analyzing the results of the differences between groups adherence to the MD regarding body composition, a greater percentage of body fat was detected in the girl population in each MD group. Similar results have been previously reported [4,37]. It might be attributed to a sedentary lifestyle and a superior level of physical inactivity in this population [38]. Muscle mass had a higher percentage in children in each MD group. However, despite the differences in the aforementioned variables with respect to gender, percentage of body fat and percentage of muscle mass at the various levels of body composition were established in previous studies [39]. No significant differences in BMI were found between boys and girls due to daily PA that positively affects their BMI [40]. The results show how ideal adherence to the MD appears to be associated with elevated levels of cardiorespiratory fitness. Similarly, participants with high adherence to the MD had better parameters of respiratory capacity due to a better body composition and cardiorespiratory profile caused by the PA that the subject’s practice [14,16]. The results obtained in handgrip strength show differences between low MD and medium MD and between medium MD and high MD in children. The increase in BMI, percentage of fat, and muscle mass due to PA causes higher levels of strength in the handgrip test [1,41]. However, girls have almost identical values in BMI and do not obtain significant differences in the percentile of handgrip strength with respect to the MD.

Similarly, one of the strengths of this study is that it allows us to predict better performance on the 20 mSRT as the KIDMED score increases. However, KIDMED score (0–12 punctuation) did not have a clear relationship with the other PF parameters. The influence of good eating habits on the PF of the population, especially in childhood, has been previously demonstrated [42,43] and could be explained by lifestyle factors, which could interact with each other in a synergistic way to influence PA level [44,45]. In any case, early adherence to the MD is key to preventing an increased risk of cardiovascular disease, metabolic syndrome, overweight, or obesity during adulthood [46,47].

Therefore, this study identifies how an optimal adherence to the MD presents a positive relationship with various PF variables in boys and is a good predictor of cardiorespiratory fitness in both cases. This study contributes suggestions on future strategies of health and educational programs, which should be based on the prevention of different diseases such as for overweight and obesity. Therefore, it is important to promote good eating and active, healthy lifestyle habits among the new generations.

This study presents certain limitations that must be explained. First, the data collection using the KIDMED questionnaire was self-reported, which could lead to an error in the reports and to memory bias due to the nature of the study. Second, it is important to highlight that socioeconomic status can affect the acquisition of good healthy habits and adherence to the MD. Finally, although the KIDMED index is the instrument most commonly used to determine the adherence to the MD, it may have been interesting to obtain information on the frequency of consumption of certain foods characteristic of the Mediterranean pattern. 

## 5. Conclusions

These results suggest that good eating habits and the practice of physical activity and sport are associated with health benefits as well as for physical fitness, mainly in boys. Thus, it is important to develop active and healthy habits from an early age, promoting strategies, public sports policies, and public health to fight off the negative health effects of incorrect eating habits and low levels of physical fitness. 

## Figures and Tables

**Table 1:** Differences between groups adherence to the Mediterranean diet (MD).

		Low MD (*n* = 85)	Medium MD (*n* = 697)	High MD (*n* = 416)
Group	Variable	M ± SD	M ± SD	M ± SD
Boys	BMI ^1^ (kg/m^2^)	18.99 ± 3.61	19.83 ± 3.57	20.17 ± 3.87
	Fat mass (%)	21.82 ± 6.09	22.50 ± 6.62	22.46 ± 6.83
	Muscle mass (%)	73.88 ± 5.72	73.38 ± 6.24	73.43 ± 6.44
	20 mSRT (percentile)	61.31 ± 25.66	64.24 ± 24.08	66.41 ± 23.84
	Handgrip (percentile)	47.94 ± 28.43	52.01 ± 28.53	60.13 ± 29.07 ^a,b^
	CMJ (percentile)	48.82 ± 26.83	43.59 ± 26.95	40.95 ± 25.52
	FVC (l)	2.45 ± 0.99	2.81 ± 0.97 ^a^	3.10 ± 1.11 ^a,b^
	FEV_1_ (l)	2.21 ± 0.87	2.50 ± 0.85 ^a^	2.72 ± 0.92 ^a,b^
	PEF (l/s)	4.02 ± 1.48	4.70 ± 1.52 ^a^	4.90 ± 1.56 ^a^
	FEF_25–75_ (l/s)	2.57 ± 0.93	3.00 ± 1.06 ^a^	3.19 ± 1.12 ^a,b^
	FEV_1_/FVC (%)	88.80 ± 9.12	88.79 ± 8.33	87.97 ± 7.71
Girls	BMI (kg/m^2^)	19.29 ± 3.76	19.33 ± 3.83	19.72 ± 3.60
	Fat mass (%)	26.97 ± 5.56	26.46 ± 5.64	27.46 ± 6.02
	Muscle mass (%)	69.26 ± 5.26	69.71 ± 5.32	68.77 ± 5.70
	20 mSRT (percentile)	65.65 ± 23.95	72.92 ± 23.26	76.10 ± 20.12
	Handgrip (percentile)	55.00 ± 30.64	53.79 ± 30.42	53.98 ± 30.18
	CMJ (percentile)	52.67 ± 18.31	52.08 ± 26.18	49.33 ± 27.61
	FVC (l)	2.40 ± 0.77	2.42 ± 0.84	2.36 ± 0.76
	FEV_1_ (l)	2.14 ± 0.58	2.18 ± 0.73	2.14 ± 0.67
	PEF (l/s)	3.89 ± 1.10	3.97 ± 1.21	4.20 ± 1.34
	FEF_25–75_ (l/s)	2.45 ± 0.77	2.69 ± 0.90	2.85 ± 0.97
	FEV_1_/FVC (%)	88.12 ± 8.92	89.58 ± 6.42	91.17 ± 5.77

^1^ BMI, body mass index; 20 mSRT, 20 m shuttle run test; CMJ, countermovement jump; FVC, forced vital capacity; FEV_1_, forced expiratory volume; PEF, peak expiratory flow; FEF_25-75_, mean forced expiratory flow between 25% and 75% of FVC_l_. ^a^ = differences vs low MD; ^b^ = differences vs medium MD.

**Table 2:** Relationship of KIDMED score and physical fitness parameters (standard errors between brackets).

Variable	20 mSRT (min)		Handgrip (kg)		CMJ (cm)		FVC (l)	
Sex	−0.58	(0.14) **	−0.33	(0.46)	1.54	(0.36) **	0.04	(0.04)
Age (years)	0.15	(0.04) **	0.73	(0.14) **	0.55	(0.11) **	0.12	(0.01) **
Weekly PA (min/day)	0.05	(0.03)	−0.19	(0.10) *	0.17	(0.08) *	0.02	(0.01)
KIDMED score	0.06	(0.03) *	0.16	(0.08)	−0.10	(0.07)	−0.01	(0.01)
BMI	−0.09	(0.04) *	0.81	(0.11) **	−0.03	(0.10)	0.08	(0.01) **
Fat mass (%)	−0.05	(0.02) *	−0.20	(0.06) **	−0.30	(0.05) **	−0.02	(0.01) **
20 mSRT (min)	-		0.00	(0.14)	0.89	(0.10) **	0.04	(0.01) **
Handgrip (kg)	0.00	(0.01)	-		0.17	(0.03) **	0.02	(0.00) **
CMJ (cm)	0.14	(0.02) **	0.27	(0.05) **	-		0.01	(0.01) *
FVC (l)	0.43	(0.15) **	2.82	(0.46) **	0.91	(0.38) *	-	
Constant	2.25	(0.51) **	−12.24	(1.54) **	12.77	(1.18) **	−0.85	(0.15) **
R^2^	0.65		0.76		0.75		0.81	
F	104.46 **		178.25 **		170.93 **		243.52 **	

PA, physical activity; BMI, body mass index; 20 mSRT, 20 m shuttle run test; CMJ, countermovement jump; FVC, forced vital capacity. * *p* < 0.05, ** *p* < 0.01.
